# Complications of Central Venous Line Insertion: A Case Series

**DOI:** 10.31729/jnma.v64i293.9282

**Published:** 2026-01-31

**Authors:** Saurav Shrestha, Rehan Pradhan, Nikol Guragain

**Affiliations:** 1 'Department of Anaesthesia, Patan Academy of Health Sciences, Lagankhel, Lalitpur, Nepal; 2 Department of Internal Medicine, Patan Academy of Health Sciences, Lagankhel, Lalitpur, Nepal; 3 School of Medicine, Patan Academy of Health Sciences, Langankhel, Lalitpur, Nepal

**Keywords:** *central venous catheterization*, *complications*, *critically ill patients*

## Abstract

Central venous catheter (CVC) insertion is a commonly performed procedure in critically ill patients. While generally safe, it carries risks of potentially serious complications, particularly when performed without ultrasound guidance or in emergent settings. We report a series of four patients who developed uncommon but significant complications following CVC placement. The first case describes the right internal mammary artery aneurysm after malplacement of CVC line. The second case outlines the formation of sternocleidomastoid hematoma after internal jugular vein access. The third case involved a guidewire retained in the inferior venacava for over one year, discovered incidentally on imaging and removed surgically. The fourth case developed a subclavian artery pseudoaneurysm post-CVC insertion, confirmed on CT angiography and surgically managed. These cases highlights the importance of strict procedural protocols, operator awareness, use of real-time imaging, and post-insertion confirmation imaging to minimize CVC-related complications. Early identification and appropriate management can significantly reduce morbidity.

## INTRODUCTION

Central venous catheter (CVC) insertion is a fundamental procedure in critical care widely used for anything from rapid fluid resuscitation, to drug administration, to parenteral nutrition, and even for administering hemodialysis. Central lines come in different sizes, types, and sites of administration.^1^ Despite its widespread use and clinical utility, CVC placement and maintenance are associated with a substantial risk of complications.

This case series highlights the spectrum of potentially serious CVC-related complications. Routine use of ultrasound guidance, strict procedural protocols, and post-procedural imaging can reduce the risk of complications while operator vigilance and early recognition remain key to improving patient safety during central venous access.

## CASE REPORT

### Patient 1

**Figure 1:**
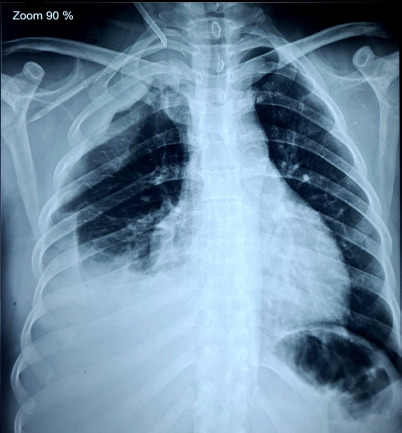
Chest radiograph showing kinking of the central venous catheter with associated rightsided hemopneumothorax following central venous catheterization (Case 1).

A 30-year-old hypertensive female, diagnosed with stage 5 Chronic Kidney Disease, presented with persistent vomiting, epigastric pain and fatigue and had flapping tremors on clinical examination. She then underwent emergency haemodialysis for uremic symptoms and was advised to continue maintenance hemodialysis. During catheter insertion over right Internal Jugular Vein (IJV), there was kinking of the catheter with malposition of the tip to the right subclavian vessel. With suspicion of arterial puncture and malplacement to the right subclavian artery, the catheter was removed. She developed right sided hemopneumothorax post-procedure and was managed by chest tube and pleurodesis. Computed Tomography (CT) scan of the chest revealed an aneurysm of the right internal mammary artery. The patient’s family denied further intervention due to financial constraints and she was discharged on regular hemodialysis. (Figure 1).

### Patient 2

A 48-year-old male, a known case of End Stage Kidney Disease was under maintenance hemodialysis. He underwent insertion of left IJV catheter after his left arteriovenous (AV) fistula had failed and right AV fistula was yet to mature. After six hours of procedure, he presented with rapidly increasing left sided neck swelling which started after bouts of cough. There was blood oozing out from the insertion site and bluish discoloration of the underlying skin. An ultrasonogram of the neck was done urgently which revealed hematoma in sternocleidomastoid with no tracheal or vascular compression. He was admitted for observation of neck swelling and the neck circumference was measured. His swelling gradually resolved and his hospital stay was uneventful. The catheter was removed after his new AV fistula matured.

### Patient 3

**Figure 2:**
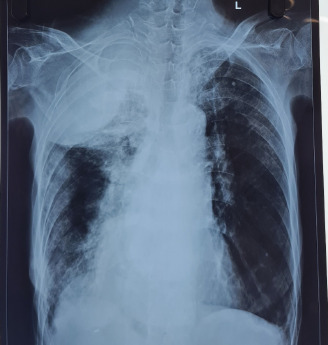
Chest radiographs of the same patient taken a year apart, both demonstrating the presence of a retained guidewire following central venous catheterization (Case 3)

A 62-year-old female, known case of Chronic Obstructive Pulmonary Disease (COPD) was under domiciliary oxygen for the past 4 months. She had the history of Pulmonary Tuberculosis 4 years back and had completed her anti-tubercular therapy. She presented to the emergency department with complaints of increased shortness of breath and increased sputum production for one day. Her chest X-ray showed a probable guidewire in situ in her inferior vena cava. On further conversation, she gave the history of hospital admission one year back in another centre and insertion of central venous catheter at that time. She was consulted with cardiothoracic and vascular surgery and was referred for removal of the guide wire after consultation. She was referred back to our centre where she completed her intravenous antibiotics along with other supportive care for her infective exacerbation and her further hospital stay was uneventful. (Figure 2)

### Patient 4

**Figure 3:**
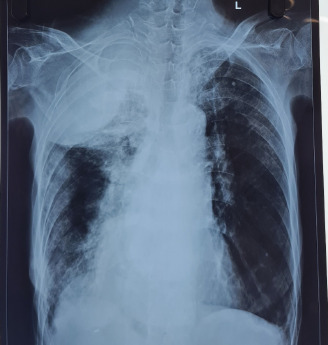
Chest radiograph of a patient who developed a pseudoaneurysm and hemothorax following central venous catheterization (Case 4).

A 80 year old female, known case of Chronic Obstructive Pulmonary Disease was under domiciliary oxygen for six years. She presented with increased shortness of breath and cough for 2 weeks. She also complained of decreased urine output for 3 days. On examination, flapping tremors were present. Her infective exacerbation of COPD was complicated by acute kidney injury with hyperkalemia refractory to medical management. Subsequently, she was advised for emergency haemodialysis and subsequently right internal jugular vein catheterization was attempted. However, due to her agitated behaviour during the catheter insertion, IJV catheterization was complicated with post-procedural hemothorax. She underwent two sessions of haemodialysis via femoral vein catheterization. CT angiography was done which showed subclavian artery pseudoaneurysm and bleeding at the origin of vertebral arteries. Surgical consultation was done and the patient was referred to another hospital with cardiothoracic surgical facilities for possible need of surgical intervention. (Figure 3)

## DISCUSSION

Central venous catheterization is an essential procedure but is inherently associated with a spectrum of potentially serious complications. The complications are divided into immediate insertion related events including arterial puncture or cannulation, hematoma, pneumothorax, hemothorax, cardiac arrhythmias, malposition, air embolism, rarely cardiac temponade and delayed catheter related complications including bloodstream infections, thrombotic events, malposition or soft tissue complications.^2^

In our first case, we reported a case of inadvertent right subclavian arterial cannulation with right internal mammary artery aneurysm and hemopneumothorax despite landmark-based catheter insertion and ultrasound assistance. While a systematic review and meta-analysis on complication rates of CVC reported that 20.4 events per 1000 catheters were placement failure and 4.4 events per 1000 catheter were associated with pneumothorax,^3^ the occurrence of internal mammary aneurysm is a rare incident and unique to our case with only few case reports reporting pseudo-aneurysms and false aneurysms.^4,5^ Understanding the Seldinger method and the factors that predispose to complications is essential for safe and effective central venous access.

In our second case, the patient developed sternocleidomastoid hematoma that developed acutely after IJV catheter insertions. Vascular injury during central venous catheterization, particularly arterial puncture, has been reported in up to 8.71% of cases, and resultant neck hematomas in nearly 6.64% of procedures, especially when landmark-based techniques are used and multiple attempts are made during emergencies.^6^ For this reason, ultrasound guidance is strongly recommended, allowing real-time visualization of the vein, surrounding vessels, and muscular planes, reducing the risk of inadvertent arterial puncture or intramuscular hemorrhage.^7^

In our third case, a retained guidewire in the inferior vena cava (IVC) was discovered one year after the central venous catheterization, incidentally during imaging for chronic respiratory symptoms. There have been numerous reports where guidewires have remained within vascular structures for extended periods of time before detection.^8^ Only 11% of retained guidewires are identified during the procedure itself, with the remainder identified during equipment clear-up (6%), after the procedure (4%), at the first check radiograph (23%), or after the first radiograph (55%).^9^ Several complications are reported with retained guidewires related to CVC placement including cardiac conduction abnormalities and dysrhythmias, thrombosis, perforation of vessels or cardiac chambers, kinking, looping or knotting of wire, breakage of distal tip and its embolism or complete loss of guidewire within the vascular system.^10^

In our fourth case, the patient developed a subclavian artery pseudoaneurysm after a failed IJV catheterization attempt. Subclavian artery pseudoaneurysm is particularly rare due to the vessel’s deep, protected anatomical location. When they occur, they are usually the result of inadvertent arterial puncture during attempts at internal jugular or subclavian cannulation, especially in uncooperative or agitated patients where repeated needle passes increase the risk of arterial trauma.^11^ COPD patients, such as in our case, may present with hyperinflated lungs that alter the usual anatomical landmarks, while agitation can lead to needle misdirection. Treatment options include open surgical resection and vascular reconstruction, endovascular exclusion, stent graft implantation and ultrasound-guided thrombin injection. When compressive symptoms exist, an open approach is advised.^12^

These complications are mostly preventable by following standard procedural protocol, adhering to correct safety measurements, and documenting the removal of guide wire after the procedure.^10^ Procedural checklists, mandatory training in central venous access for junior doctors, and supervision during early practice will further enhance safety.

## CONCLUSION

Central venous catheterization remains an essential procedure in acute and critical care, yet it carries a significant risk of complications that can be both immediate and delayed. Our series highlights the importance of vigilance throughout the procedure. Ultrasound guidance needs to be the standard of care for central venous catheterization to minimize vascular and thoracic complications. Strict observation of Seldinger's technique, with ongoing attention to guidewire control, will prevent unnecessary complications such as guidewire retention. Early detection and prompt treatment of complications, for example, referral to endovascular or surgical centers when necessary, enhance patient outcome significantly.
